# Recent Progress in the Discovery and Development of 2-Nitroimidazooxazines and 6-Nitroimidazooxazoles to Treat Tuberculosis and Neglected Tropical Diseases †

**DOI:** 10.3390/molecules25184137

**Published:** 2020-09-10

**Authors:** Hollis D. Showalter

**Affiliations:** Department of Medicinal Chemistry, College of Pharmacy, University of Michigan, Ann Arbor, MI 48109, USA; showalh@umich.edu

**Keywords:** tuberculosis, neglected tropical diseases, pretomanid, delamanid, 2-nitroimidazooxazines, 6-nitroimidazooxazoles, structure-activity relationships, chemical synthesis

## Abstract

Nitroimidazole drugs have a long history as therapeutic agents to treat bacterial and parasitic diseases. The discovery in 1989 of a bicyclic nitroimidazole lead, displaying in vitro and in vivo antitubercular activity, spurred intensive exploration of this and related scaffolds, which led to the regulatory approval of pretomanid and delamanid as a new class of tuberculosis drugs. Much of the discovery work related to this took place over a 20-year period ending in 2010, which is covered in a number of cited reviews. This review highlights subsequent research published over the 2011–August 2020 timeframe, and captures detailed structure–activity relationship studies and synthetic strategies directed towards uncovering newer generation drugs for both tuberculosis and selected neglected tropical diseases. Additionally, this review presents in silico calculations relating to the drug-like properties of lead compounds and clinical agents, as well as chemical development and manufacturing processes toward providing bulk drug supplies.

## 1. Introduction

Tuberculosis (TB) and a number of tropical diseases place a heavy economic and health burden on less developed countries, as current therapies are old and increasingly ineffective. The World Health Organization (WHO) estimates that in 2018, about 23% of the global population was infected with *Mycobacterium tuberculosis* (*M.tb*), the bacterium that causes TB [1]. According to the WHO report, a relatively small proportion (5–10%) of the estimated 1.7 billion people infected with *M.tb* will develop active TB disease during their lifetime. An estimated 10 million people fell ill with active disease in 2018, a number that has remained relatively stable in recent years. The probability of developing TB disease is much higher among people living with HIV and other risk factors, such as undernutrition and diabetes. Globally, there were an estimated 1.2 million TB deaths among HIV-negative people in 2018, and an additional 251,000 deaths among HIV-positive people. Without treatment, the mortality rate from TB is high. The currently recommended treatment for cases of drug-susceptible TB disease is a 6-month regimen of four first-line drugs: isoniazid, rifampin, ethambutol and pyrazinamide. However, a major problem associated with this regimen is the emergence of multi-drug resistance tuberculosis (MDR-TB), which is defined as resistance to isoniazid and rifampin. Treatment for people with MDR-TB is longer, and requires second-line drugs that are more expensive and more toxic. The latest data reported to WHO show a treatment success rate for MDR-TB of 56% globally. An even more ominous threat is the emergence of extensively drug-resistant TB (XDR-TB) that is resistant to these second-line drugs. The synergy of TB with HIV/AIDS and the ongoing spread of often undiagnosed MDR- and XDR-TB clearly point to the need for the development of novel drugs that act through new biological mechanisms of action to effectively treat drug-susceptible and -resistant strains [2,3]. The goal is for shorter- and easier-to-administer regimens, thus minimizing the potential for further resistance [4].

Neglected tropical diseases (NTDs) are a group of parasitic and bacterial diseases that cause substantial illness and suffering for more than one billion people globally, resulting in more than half a million deaths annually. These affect the world’s poorest people, trapping them in a cycle of poverty and disease [5]. Besides the more commonly known diseases of TB, HIV and malaria, NTDs include Chagas disease (or American trypanosomiasis), African sleeping sickness (or human African trypanosomiasis; HAT) and visceral leishmaniasis (VL), which is caused by over 20 species of the kinetoplastid protozoan *Leishmania*. Chagas disease affects people primarily in Latin America, with current therapeutic options limited to two drugs (benznidazole and nifurtimox) that can cause serious side effects and have reduced efficacy in chronic cases [6,7]. HAT is a particularly lethal NTD that is endemic in remote sub-Saharan Africa. It arises from infection with two subspecies of the kinetoplastid parasite *Trypanosoma brucei*, which are transmitted through the bite of tsetse flies [8]. The disease often progresses to a potentially fatal stage characterized by CNS disorders before treatment requiring hospitalization is applied [9]. Current drugs for late stage HAT are costly, toxic, and increasingly less effective due to drug resistance [10,11]. New drugs have been developed to address these liabilities. Fexinidazole [12] was approved by the European Medicines Agency in 2018, and acoziborole (SCYX- 7158) [13] is in Phase II/III clinical trials by the Drugs for Neglected Diseases Initiative (DND*i*). Visceral leishmaniasis (VL) is transmitted by sand flies and is most prevalent in Brazil, Sudan, Ethiopia and the Indian subcontinent [14]. It often presents with HIV coinfection and is usually fatal within two years if left untreated [15]. None of the existing VL drugs are universally effective or free from numerous drawbacks, including toxicity, high cost and emerging resistance [16]. Due to safety and tolerability issues and the need to minimize the emergence of drug resistance, there is an urgent need to develop a pipeline of new oral agents with unique mechanisms of action to combat these and other NTDs.

**Figure 1:**
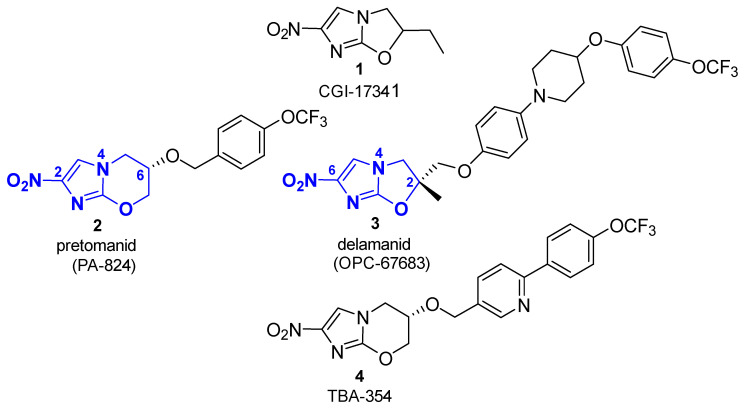
Original bicyclic imidazole lead (**1**), approved (**2**, **3**) and Phase I (**4**) drugs.

Nitroimidazole drugs have a long history as therapeutic agents to treat bacterial and parasitic diseases [17]. In 1989, researchers at Ciba-Geigy reported the discovery of the first bicyclic nitroimidazole, CGI-17341 (**1**, Figure 1), to display in vitro and in vivo antitubercular activity [18]. The compound was found to be active against drug-susceptible TB and MDR strains [19], but further development was later discontinued due to mutagenicity [19]. Nevertheless, the promising antitubercular activity spurred further structure–activity relationship (SAR) exploration of this and related scaffolds over more than a decade of intensive research. Two new bicyclic nitroimidazoles, PA-824 (**2**) [20] and OPC-67683 (**3**) [21] (Figure 1), emerged from these efforts with no evidence of mutagenicity. The further development of these followed by clinical trials resulted in the regulatory approval of pretomanid and delamanid, respectively, as a new class of TB drugs.

Pretomanid and delamanid are prodrugs that have activity against both replicating and hypoxic non-replicating mycobacteria. Under aerobic growth conditions, the drugs inhibit mycolic acid synthesis, a key reaction for cell wall formation [20,22]. Under non-replicating growth conditions, such as when cells are exposed to hypoxia, the cell wall is not actively synthesized [22]. Both effects are due to the activation of the drugs by an intracellular deazaflavin-dependent nitroreductase (Ddn). Hypoxia induces respiratory poisoning via nitric oxide release, contributing to the compounds’ bactericidal activity [23,24]. This mode of action is distinct from the postulated nucleophilic nitro reduction of other nitroaromatic TB agents to form reactive nitroso intermediates, which trigger suicide inhibition of an enzyme (DprE1) involved in cell wall biosynthesis [25,26]. The mode of action of bicyclic nitroimidazoles in *Leishmania* is different from the mode of action for *M.tb*. It has recently been discovered that bicyclic nitroimidazoles are activated by a novel nitroreductase (NTR2) in *Leishmania* [27].

Both pretomanid and delamanid are lipophilic in nature, which in fact might help entry through the highly lipophilic cell wall of *M.tb*, and be responsible for their high potency [28,29]. A serious problem with both drugs is that they are poorly water soluble [30,31], with a propensity to bind to human plasma proteins [24]. The highly lipophilic nature of these compounds presented absorption issues during clinical trials, which were managed by altering the formulation.

This review summarizes recent progress in the discovery and development of agents within the classes of 2-nitro-6,7-dihydro-5*H*-imidazo[2,1-*b*][1,3]oxazines and 6-nitro-2,3-dihydroimidazo[2,1-*b*]oxazoles (herein referred to as 2-nitroimidazooxazines and 6-nitroimidazooxazoles, respectively), to treat tuberculosis and, more recently, selected neglected tropical diseases. A cursory history is given of work leading to the discovery of pretomanid and delamanid, and early follow-on SAR studies, which captures a ~20-year period ending in 2010. This is summarized in a number of cited reviews (vide infra). The main aim of this review is to highlight subsequent research detailed in journal articles and patents over the 2011–August 2020 timeframe. These provide detailed SAR studies and synthetic strategies directed towards uncovering newer generation drugs, and chemical development processes toward providing bulk- and manufacturing-scale drug supplies for pretomanid, delamanid and other clinical candidates.

For the TB studies reported below, compounds were screened in culture against *M.tb* (strain H37Rv) in growth inhibition assays. The recorded minimum inhibition concentration (MIC) values vary slightly for a given agent depending on the assay conditions and method of measuring growth. The reported literature MIC values are for a 90–100% growth inhibitory effect. Two commonly used assays are the microplate alamar blue assay (MABA) and the low oxygen recovery assay (LORA), which are conducted under either aerobic (replicating) or hypoxic (non-replicating) conditions, respectively [32,33,34]. Given that both pretomanid and delamanid exhibit at least two distinct mechanisms of action against TB, depending on the oxygenation environment [23], the LORA assay is regarded as a useful starting tool to identify analogues that may be more efficient at killing persistent subpopulations of *M.tb* in vivo [32,33].

## 2. 2-Nitroimidazooxazines

### 2.1. Pretomanid (PA-824; **2**)

The initial work in the synthesis of pretomanid (**2**) and early analogues as antitubercular agents was carried out by the PathoGenesis Corporation. This and other discovery efforts have been reviewed in a 2010 paper by Denny and Palmer [35], in which they highlight synthetic [36,37] and SAR studies [37,38,39] leading to pretomanid, and present follow-on work toward uncovering second-generation analogues [40,41,42]. Another comprehensive review in 2011 by Mukherjee and Boshoff [43] summarizes medicinal chemistry studies leading to the discovery of pretomanid, its advancement to new drug approval (NDA) status [37,40,41,44,45], and follow-on SAR studies toward potential alternatives for further development [42,46,47]. Several tables provide a detailed listing of analogues and a graphic summarizes SAR trends. One superior candidate, TBA-354 (**4**, Figure 1), emerged from these studies [47]. It displayed lower MICs than pretomanid, stability in human and mouse liver microsomes, good bioavailability in rats, and outperformed both pretomanid and delamanid in vivo in a number of murine infection models of TB [48]. It was advanced to a Phase I clinical trial, but the study was halted in early 2016 due to side effects in the initial cohort of healthy volunteers [49,50].

Pretomanid received regulatory approval in the United States in 2019 as part of a combination regimen with bedaquiline and linezolid for the treatment of adults with pulmonary XDR-TB or for treatment-intolerant or nonresponsive MDR-TB [51].

### 2.2. Pretomanid (2-Nitroimidazooxazine) Analogues

**Figure 2:**
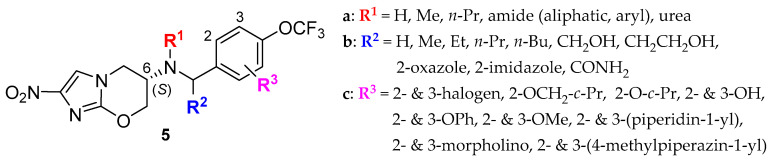
6-(*S*)-Aminopretomanid analogues **5** with variations at R^1^, R^2^, and R^3^.

Cherian et al. applied a previously derived 3D-quantitative structure–activity relationship (QSAR) pharmacophore model [41], with only one hydrophobic side chain feature, which was reasonably predictive for aerobic MIC activity, to prepare a large series of analogues of pretomanid (**2**) with an additional distinct hydrophobic feature in its tail region [52]. To this end, three subseries were synthesized, with the first (**5a**: R^1^) employing the more soluble 6-(*S*)-amine as an attachment site, the second (**5b**: R^2^) focusing on the benzylic carbon, and the third (**5c**: R^3^) exploring additional diversity on the aromatic nucleus of the 4-(trifluoromethoxy)benzyl moiety of the parent compound (Figure 2). Compounds were tested for whole-cell activity against both replicating and non-replicating *M.tb*. Two of the three positions (R^1^ and R^3^) provided substantial SAR and improved compounds in terms of both potency and solubility, while one site (R^2^) provided little useful SAR. Additionally, while C-6 (*S*)-stereochemistry is required, potency is independent of the configuration at the benzylic carbon substituent R^2^. In general, the highest potency for R^1^ was exhibited by small alkyl substituents. Variations at R^3^ provided the greatest potency enhancements, with identical 2- and 3-position mono substituents exhibiting similar activity. The best compounds within the R^3^ subseries (wherein R^1^, R^2^ = H) showed MICs of 0.06–0.10 µM aerobic and 2.0–4.4 µM anaerobic whole cell activities, compared to 0.63 μM and 8.8 μM, respectively, for pretomanid. In addition, the kinetic parameters of substrates of the deazaflavin-dependent nitroreductase (Ddn) from *M.tb* that reductively activates these pro-drugs were determined. Some of these compounds also exhibited enhanced solubility, with acceptable stability to microsomal and in vivo metabolism.

**Figure 3:**
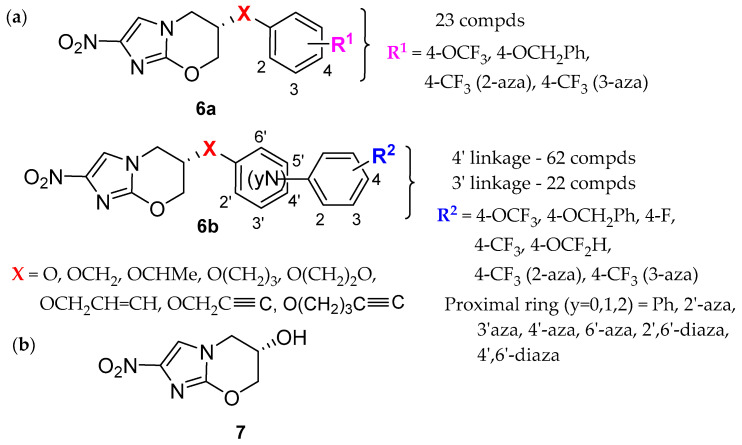
(**a**) Subseries (**6a**, **6b**) of novel pretomanid analogues with variable ether linkers (X); (**b**) Toxic metabolite **7**.

In an ongoing collaboration between the Global Alliance for TB Drug Development and the Auckland Cancer Society Research Centre in New Zealand, Thompson et al. presented two subseries of 107 new analogues (**6a**, **6b**; Figure 3a) of pretomanid, which featured alternative side chain ether linkers of varying size and flexibility toward seeking compounds with enhanced metabolic stability and high efficacy in mouse models [53]. With respect to SAR trends, both X = O and OCHMe (as diastereomeric mixtures) were broadly tolerated in vitro, with an example in the latter subseries (**6b**; X = O, 4′-linked biphenyl, R^2^ = 4-OCF_3_; MABA = 0.09 µM and LORA = 1.1 µM) exhibiting excellent pharmacokinetics in plasma and lung tissue following oral dosing, and an eight-fold better efficacy than pretomanid in a mouse model of acute *M.tb* infection. This compound also displayed negligible fragmentation when exposed to bicyclic alcohol metabolite **7** (Figure 3b) in liver microsomes, which, if present, could signal potential mutagenicity based on Ames data reported for structurally related 6-nitroimidazooxazoles [54]. Extended linkers provided greater potencies against replicating *M.tb* (for monoaryl analogues **6a**), with propynyl ethers being most effective under anaerobic (non-replicating) conditions (for both mono and biaryl analogues). For benzyloxybenzyl and biaryl derivatives, aerobic activity was maximal with the original OCH_2_ linker. One compound (**6b**; X = oxypropynyl, 4′-linked biphenyl, R^2^ = OCF_3_) displayed an 89-fold higher efficacy than pretomanid in an acute mouse model of infection, and was slightly superior to delamanid in a chronic model. More soluble pyridine/pyrimidine analogues within the **6b** subseries were less effective. Both of the in vivo efficacious compounds cited above displayed poor solubility (< 0.5 µg/mL in water at pH 7) and thus were not advanced further.

**Figure 4:**
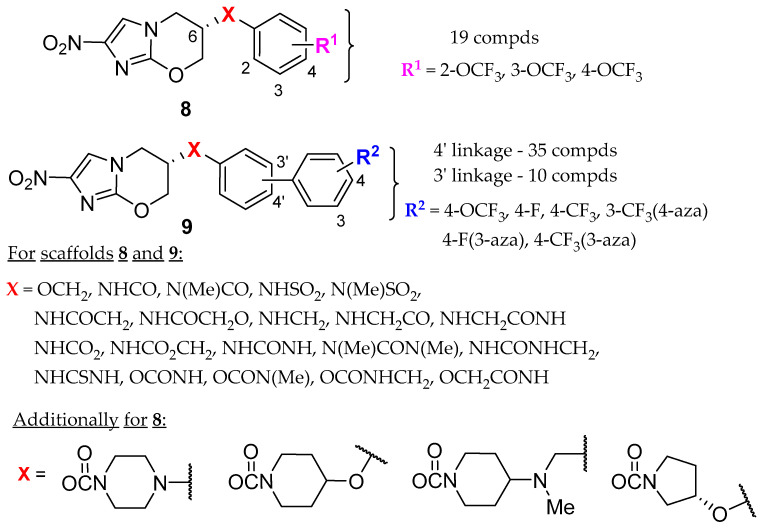
Analogues of pretomanid with variable linkers (X) for scaffolds **8** and **9**.

**Figure 5:**
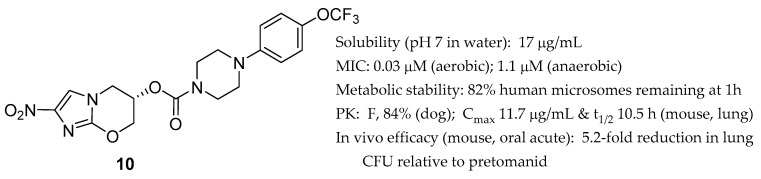
Profile of potential development compound **10**.

With the backdrop of these studies, a follow-up campaign was carried out by the New Zealand group to explore additional linker options considered to better address metabolic stability [55]. Their strategy was to replace the OCH_2_ linkage of pretomanid with various amide, carbamate and urea functionalities, and evaluate these analogues via their ability to circumvent oxidative metabolism, reduce compound lipophilicity and improve aqueous solubility (while maintaining reasonable in vivo efficacy) in order to derive a superior TB drug candidate with enhanced safety. The genera of 68 synthesized novel compounds are shown in Figure 4, featuring the 2-nitroimidazooxazine core of pretomanid with two principal variations (monoaryl, **8**; biaryl, **9**) off the 6-position linker (X). Several soluble monoaryl examples (e.g., **8**: X = NHCOCH_2_ and NHCOCH_2_O; R^2^ = 4-OCF_3_) displayed moderately improved (approximately two- to four-fold) potencies against replicating *M.tb* relative to pretomanid, but were generally inferior inhibitors under anaerobic (non-replicating) conditions. More lipophilic biaryl derivatives (e.g., **9**: X = NHCOCH_2_ and NHCOCH_2_O, Ph-4′-Ph, R^2^ = 4-OCF_3_) mostly displayed similar or reduced potencies in these assays in contrast to the parent biaryl congener (**9**; Ph-4′-Ph, R^2^ = 4-OCF_3_) with the X = OCH_2_ linker. All were poorly soluble. The leading biaryl carbamate (**9**; X = OCONH, Ph-4′-Ph, R^2^ = 4-OCF_3_) demonstrated exceptional metabolic stability and a five-fold better efficacy than pretomanid in a mouse model of acute *M.tb* infection, but was poorly soluble. Bioisosteric replacement of this biaryl moiety of **9** by an arylpiperazine [56] resulted in a soluble, orally bioavailable carbamate analogue (**10**; Figure 5), providing superior activity to pretomanid and identical activity to delamanid in a mouse acute infection model, and comparable efficacy to delamanid in a chronic model. The compound displayed a favorable pharmacokinetic profile across several species, and enhanced safety as assessed by no mutagenic effects in an Ames test conducted with and without metabolic activation. The compound also showed very low inhibition of hERG (IC_50_ > 30 μM). Representative data are shown in Figure 5. Despite its favorable profile for potential development, no follow-up studies have been reported on this compound.

**Figure 6:**
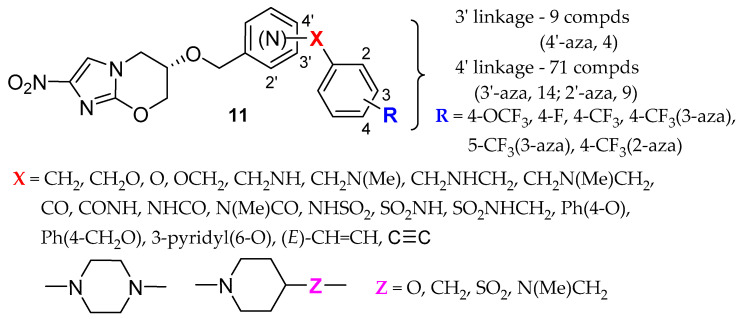
Analogues (**11**) of pretomanid with variable linkers (X) between aryl rings.

**Figure 7:**
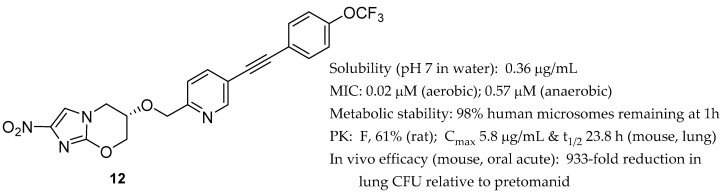
Profile of potential development compound **12**.

A second follow-up campaign was carried out to examine novel extended side chain 2-nitroimidazooxazine analogues (**11**, Figure 6), featuring diverse linker groups (X) between two aryl rings as a potential strategy to improve solubility and oral activity against chronic infection by *M.tb* [57]. The 80 novel synthesized analogues featured both lipophilic and highly polar functionalities (e.g., X = carboxamide, alkylamine, piperazine, piperidine). These were well tolerated in vitro (except for sulfonamides), and the hydrophilic linkers provided some solubility improvements, particularly in combination with pyridine rings. A subset of compounds (4′-linked) was further evaluated and showed high microsomal stabilities, although in the acute infection mouse model, just one biphenyl (**11**; X = (*E*)-ethene, 4-OCF_3_; six-fold) and two pyridine-phenyl linked derivatives (**11**: X = ethyne, 3-aza, 4-CF_3_; five-fold and **12**: X = ethyne, 2′-aza, 4-OCF_3_; > 933-fold) gave in vivo efficacies notably superior to pretomanid. Compound **12** also displayed outstanding in vivo activity in the stringent chronic model (up to 24-fold better than delamanid) with favorable pharmacokinetics, including good oral bioavailability in the rat. Representative data for **12** is summarized in Figure 7. The compound showed no mutagenicity in the Ames test and exhibited a negligible time-dependent release of alcohol metabolite **7**, implicated in earlier studies as leading to toxicity [53], following HLM and MLM incubations. It also provided a good therapeutic index toward inhibition of hERG. However, the low aqueous solubility of **12** and its greater binding (99%) to human plasma proteins rendered this compound less attractive than the earlier clinical trial agent TBA-354 (**4**, Figure 1), and thus it was not further pursued toward development.

**Figure 8:**
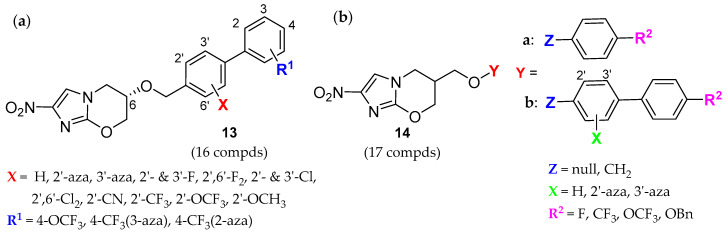
(**a**) biaryl -OCH_2_- (**13**) and (**b**) -CH_2_O- (**14a**,**b**) analogues to study effects on oxidative metabolism.

In ongoing efforts toward identifying a backup to pretomanid, Thompson et al. earlier observed that certain biaryl analogues displayed enhanced in vivo efficacies, yet retained some susceptibility towards oxidative metabolism thought to be associated with an increased toxicity risk [54] (*vide supra*). To further explore this, a new strategy was investigated via two modifications of the 6-position substituent off the 2-nitroimidazooxazine core (Figure 8) [58]. First, a series of 16 novel biaryl analogues (**13**; Figure 8a) employing the OCH_2_ linker was investigated. Ortho-substitution of the proximal aryl ring with larger electron-withdrawing substituents (e.g., **13**; X = 2′,6′-Cl_2_, 2′-CF_3_, 2′-OCF_3_) maintained or improved compound stability but reduced aerobic potency. However, X = 2′-F and 2′-CN maintained or increased potency, but with lowered stability. In vivo, only two novel analogues (**13**; X = 2′- or 3′-F; R = 4-OCF_3_) preserved high efficacy against an acute infection mouse model (33- and > 933-fold, respectively, relative to pretomanid), with the former superior to pretomanid (16-fold) and delamanid (2.2-fold) against a chronic model. Since the metabolic stability of biaryl analogues **13** with proximal ring substitution achieved only limited success, reversal of the 6-oxymethylene linkage (**14a**, monoaryl and **14b**, biaryl; Figure 8b) was examined with 17 novel analogues. Several compounds displayed high aerobic potency and selected ones improved stability towards human liver microsomes. However, the in vivo results were inferior. The findings of this study augmented earlier SAR studies regarding metabolic stability, suggesting that previous lead compounds such as **4** [47] and two related acetylene-extended analogues (e.g., **6b**; X = OCH_2_-ethyne, 4′-linked biphenyl, R^2^ = 4-OCF_3_ [53] and **12** [57]) were already ideally positioned to maximize binding interactions with the known deazaflavin-dependent nitroreductase Ddn [59]. This, in conjunction with their more suitable pharmacokinetic properties, which facilitated essentially optimal in vivo activity, led the New Zealand group to conclude that various alternative SAR directions in the 6-substituted 2-nitroimidazooxazine class would likely be unable to yield either comparable or superior results.

**Scheme 1:**
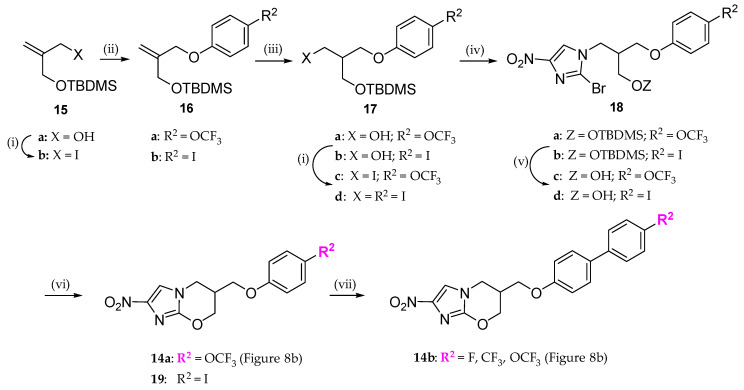
(**i**) I_2_, PPh_3_, imidazole, DCM, 5–20 °C; (**ii**) R-PhOH, K_2_CO_3_, acetone, 50 °C; (**iii**) I_2_, NaBH_4_, THF, 0–20 °C, then 30% H_2_O_2_, 3 N NaOH, 0–20 °C; (**iv**) 2-bromo-4-nitro-1*H*-imidazole, K_2_CO_3_, DMF, 85 °C; (**v**) 1% HCl in 95% EtOH, 20 °C; (**vi**) NaH, DMF, 0–20 °C; (**vii**) for **19**: ArB(OH)_2_, 2M aq Na_2_CO_3_, toluene, EtOH, (DMF), Pd(dppf)Cl_2_, 90 °C.

The synthesis of OCH_2_ linker analogues **13** shown in Figure 8a employed standard methodologies, starting from bicyclic alcohol **7**, as previously outlined [37,42]. However, the synthesis of the more complex reverse linker analogues **14a**,**b** (Figure 8b) required the development of new routes. One of these is shown in Scheme 1. Thus, the alkylation of 4-substituted phenols (R = OCF_3_, I) with known iodide **15b** [60] gave phenolic ethers **16a**,**b**. Hydroboration of the terminal double bond provided the intermediate alcohols **17a**,**b**. The conversion of these to iodides **17c**,**d**, followed by alkylation of 2-bromo-4-nitro-1*H*-imidazole by each of these, gave derived *tert*-butyldimethylsilyl (TBDMS) ethers **18a**,**b**, respectively. TBDMS ether deprotection gave alcohols **18c**,**d**, which were ring-closed with NaH to give racemic bicyclic target compound **14a** and iodide **19**, respectively. The latter was further elaborated via Suzuki couplings to the biaryl derivatives **14b**. Racemic **14a** (R^2^ = OCF_3_) was separated into its two enantiomers via preparative chiral HPLC. The *R*-enantiomer has an MIC of 0.23 µM (MABA) and 1.9 µM (LORA), 2.2- and 4.7-fold more potent than the *S*-enantiomer, respectively. Racemic compound **14b** (R = OCF_3_) has MIC values of 0.14 µM (MABA) and >128 µM (LORA). To date, there has been no reported synthesis of this bicyclic core with fixed chirality off the 6-position.

**Figure 9:**
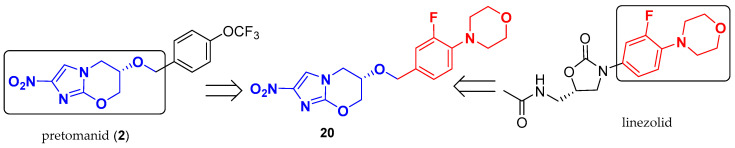
Hybrid analogue (**20**) of pretomanid (**2**) and linezolid.

**Scheme 2:**
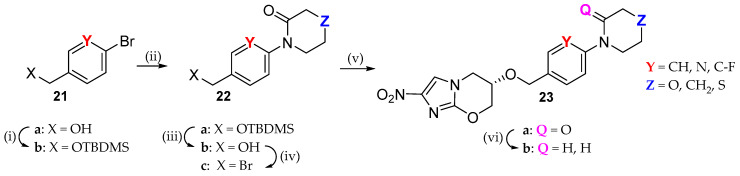
(**i**) TBDMSCl, imidazole, DCM, rt; (**ii**) six-membered ring lactam, *N*,*N*’-dimethylethylenediamine, K_2_CO_3_, CuI, toluene, reflux; (**iii**) TBAF, THF, rt; (**iv**) Ph_3_P, CBr_4_, DCM, rt; (**v**) (*S*)-2-nitroimidazooxazin-6-ol (**7**), NaH, DMF, rt; (**vi**) BH_3_ · Me_2_S, THF, rt.

The poor aqueous solubility and high protein binding of pretomanid presents opportunities for improvement in its physiochemical properties. Lee et al. [61] attempted to address these liabilities by synthesizing a series of hybrid compounds that draw on the excellent physicochemical properties of the oxazolidinone linezolid, which has shown impressive activity for the treatment of drug-resistant tuberculosis [62]. Their approach was to incorporate the outer ring elements found in first and second generation oxazolidinones into the 2-nitroimidazole core of pretomanid (**2**), to improve its physicochemical and antitubercular properties. The strategy as applied to the direct hybrid (**20**) of pretomanid and linezolid is shown in Figure 9. The synthetic approach successfully produced 17 hybrids (**23**) that maintained antitubercular activity and had improved physicochemical properties (Scheme 2). The hybrid (**20**) displayed MIC (aerobic conditions) similar to pretomanid (0.78 vs. 0.39 µg/mL), comparable aqueous solubility (10.1 vs. 8.5 µg/mL, pH 7.4) and stability in mouse microsomes (t_1/2_ 7.7 h vs. 8.1 h), and much improved protein binding (51% vs. 93%), respectively. With respect to PK, its clearance (16.5 mL/min/kg) and volume of distribution (1.2 L/kg) were close to those of pretomanid (12.1 mL/min/kg and 1.6 L/kg, respectively). However, **20** and two other advanced analogues that were evaluated in a chronic mouse model of tuberculosis infection surprisingly lacked efficacy. This suggests that the tail portion of pretomanid, not found in the hybrid molecules, is required for in vivo efficacy.

**Scheme 3:**
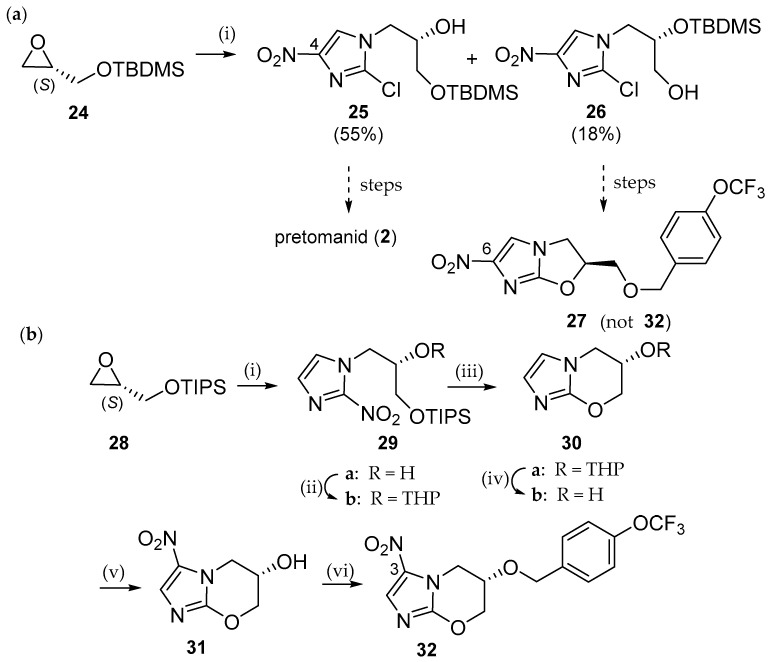
(**a**) and (**b**): (**i**) 2-chloro-4-nitro-1*H*-imidazole, K_2_CO_3_, EtOH, 80 °C. (**b**): (**ii**) 3,4-dihydro-2*H*-pyran, PPTS, toluene, 20 °C; (**iii**) TBAF, THF, 124 °C (sealed tube); (**iv**) conc. HCl, MeOH, 20 °C; (**v**) TFAA, −5 to 0 °C, then conc. HNO_3_, −50 to 20 °C; (**vi**) 4-(trifluoromethoxy)benzyl bromide, NaH, DMF, 0–20 °C.

In an earlier publication detailing a synthesis of pretomanid, a side product was isolated and assigned as its 3-nitro isomer (**32**; Scheme 3b) [41]. The abbreviated original synthetic pathway is shown in Scheme 3a. Recently, Thompson et al. reexamined this synthesis following an analysis of a trace by-product, also assigned as the 3-nitro isomer, that had been isolated from early process optimization studies toward a large-scale synthesis of pretomanid [63]. The NMR of the assigned 3-nitro isomer from each lab did not match, thus stimulating further investigation. Thus, the reaction of 2-chloro-4-nitro-1*H*-imidazole with the TBDMS ether derivative of *R*-glycidol **24** was rerun, which gave two products in a ~3:1 ratio, with the major one being the expected 4-nitroimidazole derivative **25**, as previously described. However, the minor isomer was determined to be **26** by rigorous NMR experimentation, which is ascribed to the migration of the TBDMS group of **25** to give secondary ether **26** under the basic reaction conditions, and not from attack of epoxide **24** by the alternate imidazole nitrogen. The further reaction of ether **26** under the conditions described would have led to bicyclic 6-nitroimidazooxazole **27**, not **32** as assigned. Following this discovery, the New Zealand group developed a de novo synthesis of authentic **32**, as shown in Scheme 3b. The six-step synthesis resulted in 17% overall yield and utilized the more stable triisopropylsilyl (TIPS) protecting group on starting epoxide **28**. Nitration of the chiral des-nitro imidazooxazine alcohol **30b** in trifluoroacetic anhydride gave intermediate **31**, onto which the side chain was attached to provide **32**. The structure of **32** was verified by x-ray crystallography. Compound **32** displayed no antitubercular activity (MIC > 128 µM).

**Figure 10:**
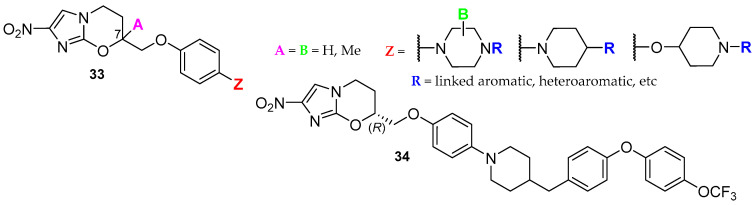
Genus of compounds (**33**) with transposed side chains and most potent analogue (**34**).

A patent from the Otsuka Pharmaceutical group in Japan discloses the synthesis of a large series of compounds in which the C-6 side chain found in pretomanid and analogues has been transposed to the C-7 position of the bicyclic 2-nitroimidazooxazine core, along with an inversion of the -OCH_2_ linker moiety [64]. The 772 named compounds **33** are generically captured in Figure 10, and include subsets that are C-7 racemates or possess *R*- or *S*-stereochemistry. From the 540 compounds with experimental details, MICs vs. *M*.*tb* in 7H11 medium were generated for 34 representatives of these subsets, and range from 0.1 to ≤ 0.0004 µg/mL for the most potent compound **34**.

**Scheme 4:**
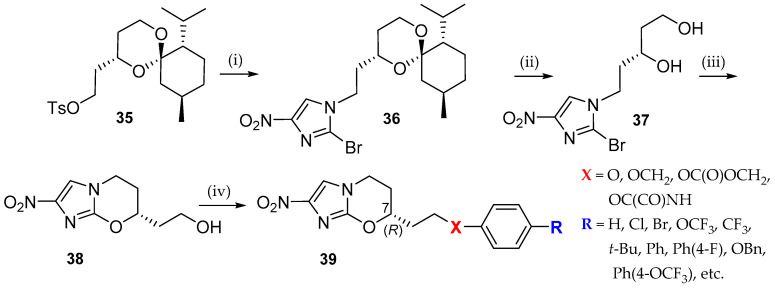
(**i**) 2-bromo-4-nitro-1*H*-imidazole, K_2_CO_3_, cat. TBAI, DMF, 120 °C; (**ii**) Dowex 50Wx8 resin (H^+^), MeOH, rt; (**iii**) Cs_2_CO_3_, DMF, 75 °C; (**iv**) (**a**) R-PhOH, PPh_3_, DIAD, DMF, 0 °C–rt; (**b**) R-BnBr, NaH, DMF, −60 °C to rt; (**c**) R-BnBr, Ag_2_CO_3_, cat. TBAI, DMF, 75 °C; (**d**) R-PhNCO, cat. CuCl, DMF, rt.

Building on this work, Kang et al. reported on a related series of compounds with an extended ethoxy linker (Scheme 4) [65]. This extended linker to give **39** was expected to increase the conformational mobility of the 4-(trifluoromethoxy)benzyl group of pretomanid, which in turn could affect solubility through the inhibition of pi–pi stacking observed in its crystal structure [44]. The new analogues contain C-7 benzyl ether, phenyl ether, benzyl carbonate and phenyl carbamate fragments, with a range of mono substituents off the 4-positions of the phenyl moiety. Starting from the known chiral spiroketal tosylate **35** [66], coupling with 2-bromo-4-nitro-1*H*-imidazole provided intermediate **36**, which was transformed to key intermediate **38** in two routine steps. This was then coupled via standard methodologies to provide a range of analogues **39**.

Among the 23 synthesized compounds screened for in vitro activity against *M.tb*, most exhibited submicromolar potency and low toxicity (TC_50_ > 100 µM), with the best compound (**39**, X = O, R = Ph(4-F) giving MIC 0.050 µM. The closest congener (X = OCH_2_; R = OCF_3_) to pretomanid gave MIC 0.078 µM, compared to 0.390 µM for its (*S*)-enantiomer and pretomanid. No measured solubility data are presented in support of the design rationale.

**Figure 11:**
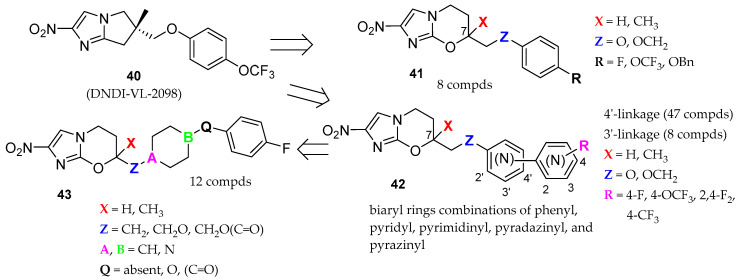
Genus of analogues **41**–**43** for treatment of visceral leishmaniasis.

**Scheme 5:**
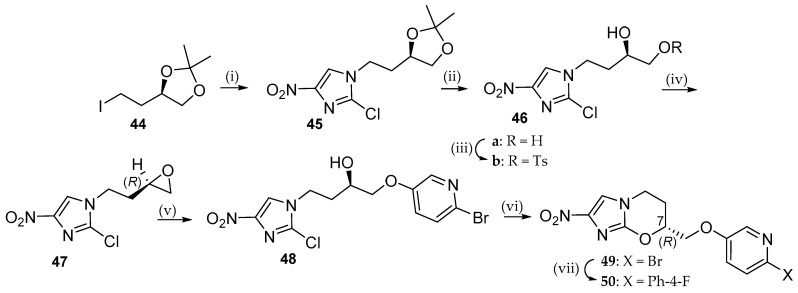
(**i**) 2-chloro-4-nitro-1*H*-imidazole, K_2_CO_3_, DMF, 70 °C; (**ii**) 1 M HCl, MeOH, 0–20 °C; (**iii**) TsCl, pyridine, −10 to 20 °C; (**iv**) DBU, CH_2_Cl_2_, 0–20 °C; (**v**) 2-Br-5-OH-pyridine, K_2_CO_3_, MEK, 84 °C; (**vi**) NaH, DMF, 0–20 °C; (**vii**) 4-F-PhB(OH)_2_, DMF, toluene, EtOH, 2 M Na_2_CO_3_, Pd(dppf)Cl_2_ under N_2_, 87 °C.

In another study related to the Otsuka work, Thompson et al. invoked a scaffold hopping approach [67] by relocating aromatic side chains from the 6-position to the 7-position of the 2-nitroimidazooxazine core, with attachment via the same inverted linker (CH_2_OR) that is also present in the 6-nitroimidazooxazole compound DNDI-VL-2098 (**40**, Figure 11) [68]. This compound, discovered by phenotypic screening by the Drugs for Neglected Diseases initiative (DND*i*) of some nitroimidazole derivatives arising from early studies by the New Zealand group with the TB Alliance, became a preclinical candidate against the kinetoplastid disease visceral leishmaniasis (VL). A novel nitroreductase (NTR2) has been identified as the enzyme responsible for its activation in *Leishmania* parasites [27]. The goal was to uncover efficacious backup compounds with improved safety and stability. This led to the discovery of 75 novel 7-substituted 2-nitroimidazooxazine derivatives (Figure 11). Commencing with biphenyl **42** (X = H, Z = O, Ph-4′-Ph, R = 4-F), bioisosteres formed by replacing one phenyl with pyridine or pyrimidine showed improved solubility and potency. More hydrophilic side chains such as piperazine or piperidine (**43**; X = H or Me, Z = CH_2_, A = N, B = N or CH, Q = absent) for the proximal ring of **42** produced even greater solubility enhancements, but reduced VL activity overall. In a *Leishmania donovani* mouse model, two racemic phenylpyridines (**42**; X = H, Z = O, 3′-aza, 4′-linkage, 4-F or 4-OCF_3_) were superior, with the 4-F analog providing >99% inhibition at 12.5 mg/kg (bid, orally) in the *Leishmania infantum* hamster model. Overall, the 7-*R*-enantiomer of **42** (X = H, Z = O, 3′-aza, 4′-linkage, 4-F) displayed more optimal efficacy, pharmacokinetics and safety, leading to its selection as the preferred development candidate. The compound, designated as DNDI-0690 (**50**), was nominated as a preclinical candidate in 2015, and is currently in Phase I clinical trials [69,70]. Its synthesis is shown in Scheme 5. By alkylating 2-chloro-4-nitro-1*H*-imidazole with (4*R*)-4-(2-iodoethyl)-2,2-dimethyl-1,3-dioxolane (**44**) [71], the chiral acetal product **45** was readily converted into the *R*-epoxide **47** by successive hydrolysis to diol **46a**, tosylation at the primary hydroxyl (**46b**), and internal substitution to form the oxirane ring. This was then elaborated to **50** by reaction with 2-bromo-5-hydroxypyridine to give **48**, ring closure to **49**, and Suzuki coupling.

Further, some of the best VL leads showed highly potent in vitro effects against TB, with both *R*-enantiomers and 4-trifluoromethoxy analogues most preferred as leads for potential development. The *R*-enantiomer of the racemate above (**42**; X = H, Z = O, 3′-aza, 4′-linkage, 4-OCF_3_) showed MABA MIC 0.024 µM and LORA MIC 1.5 µM vs. 0.50 µM and 2.6 µM, respectively, for pretomanid [42].

**Figure 12:**
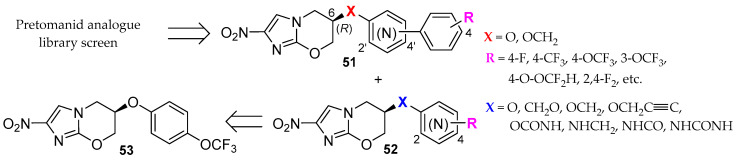
Genus of synthesized compounds (**51**–**53**) to treat visceral leishmaniasis.

The New Zealand group followed up with a second campaign to find an optimal 2-nitroimidazooxazine clinical candidate to treat VL [72]. Initial approaches to scaffold variations of pretomanid (e.g., nitroimidazooxazepines, nitrotriazolooxazines, nitropyrazolooxazines) were not fruitful. However, the screening of a 900-compound pretomanid analogue library provided several hits with more suitable potency, solubility and microsomal stability. Those giving superior efficacy were the newly synthesized 6-position *R*-enantiomers of phenylpyridine-based side chains (**51**), as established through head-to-head assessments of enantiomeric pairs in a *Leishmania donovani* mouse model (Figure 12). Two such leads (**51**, X = OCH_2_, 2′-aza, 4′-linkage, 4-OCF_3_ or 2-F/4-OCF_3_) displayed promising activity in the more stringent *Leishmania infantum* hamster model, but were unexpectedly found to be potent inhibitors of hERG.

Even though one O-linked phenylpyridine (**51**: X = O, 3′-aza, 4′-linkage, 4-OCF_3_) displayed superb activity in the mouse VL model, a decision was made to focus instead on the less lipophilic monoaryl series **52.** An extensive SAR investigation pinpointed two compounds (**52**, X = O, 2-aza, 4-CF_3_ and **53**) with superior solubility values (12–110 µg/mL) and excellent PK profiles in three species (mouse, rat and hamster), which also provided excellent oral efficacy in the chronic hamster model (>97% parasite clearance at 25 mg/kg, bid) and exhibited minimal hERG inhibition. Additional profiling earmarked **53** (DNDI-8219) as the favored backup development candidate. In total, 76 new compounds were synthesized. A small subset of racemic compounds was tested in vitro against *M.tb*, with none exhibiting exceptional activity.

**Figure 13:**
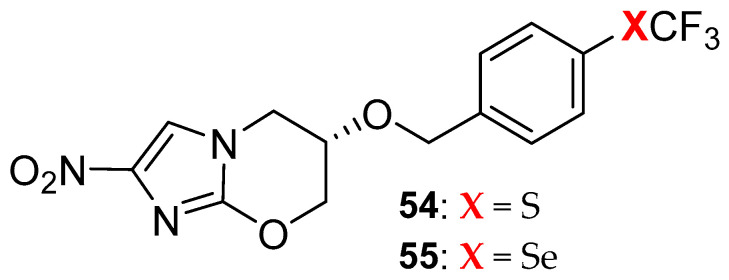
Chalcogen isosteres (**54**, **55**) of pretomanid.

A recent report outlines the synthesis of two chalcogen (SCF_3_, **54**; SeCF_3_, **55**) isosteres of pretomanid (Figure 13) [73]. The replacement of OCF_3_ with these moieties modulates physicochemical and ADME properties without introducing intrinsic liabilities. These congeners are more lipophilic (by ~0.5 logP units) and display slightly lowered aqueous solubility and passive permeability in MDCK cells than their oxygen counterpart. However, the microsomal stability in human liver microsomes was unchanged, indicating that these molecular changes may be beneficial for in vivo half-life. No microbiological data were given for either compound in this paper, nor were any given earlier in a patent application that first disclosed the synthesis of **54** [74].

## 3. 6-Nitroimidazooxazoles

### 3.1. Delamanid (OPC-67683; **3**)

Discovery work resulting in the selection of delamanid as a clinical candidate was carried out by the Otsuka Pharmaceutical Company in Japan [21,54,75,76]. SAR studies of this work are summarized in two reviews [43,77]. Successful drug development efforts resulted in the regulatory approval of delamanid by the European Medicines Agency (EMA) in 2014 for the treatment of MDR-TB [78,79].

### 3.2. Delamanid (6-Nitroimidazooxazole) Analogues

The initial work in the synthesis of delamanid and analogues as antitubercular agents was carried out by Otsuka Pharmaceutical Co. in Japan. Two patents describe the preparation of ~3400 compounds [76,80]. Selected compounds from the earlier filing, including delamanid, are profiled in detail in a 2006 publication [75]. Subsequent disclosures, from other laboratories, of additional analogues from 2015 to the present are summarized below, including campaigns toward discovering new drugs to treat kinetoplastid diseases, such as visceral leishmaniasis, and other neglected tropical diseases.

**Figure 14:**
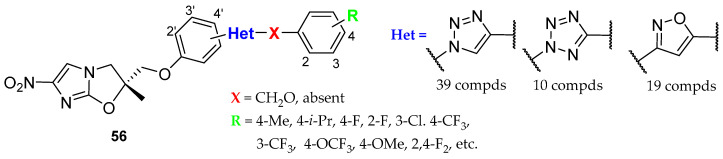
Delamanid analogues (**56**) with central piperidinyl replaced by heteroaryl moieties.

**Scheme 6:**
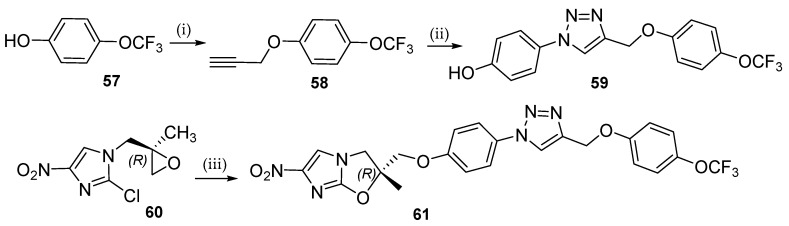
(**i**) propargyl bromide, K_2_CO_3_, CH_3_CN, rt; (**ii**) 4-azidophenol, CuSO_4_, *t*-BuOH, H_2_O, sodium ascorbate, rt; (**iii**) **59**, NaH, DMF, 0–50 °C.

A patent by an Indian group discloses the synthesis of a series of 68 novel compounds, in which the central piperidyl ring (Het) of delamanid has been replaced with triazolyl, tetrazolyl and isoxazolyl moieties (**56**; Figure 14) [81]. A follow up paper discusses the synthesis and SAR of a subset of the triazolyl and isoxazolyl classes [82]. When tested in vitro against *M.tb*, the overall screening results indicated that replacement by these heterocycles was acceptable, with further potency enhancements dependent on the presence of substitution on the distal phenyl ring. In most cases, R = F and OCF_3_ were more favorable and showed comparatively better activity. Compounds with MICs of 0.57–0.13 µM were further screened against dormant and resistant strains of *M.tb*. Based on its overall in vitro profile and favorable in vivo oral pharmacokinetics, one compound (**61**; Scheme 6) with an aerobic MIC of 0.23 µM was evaluated in efficacy studies against an intranasal mouse model of acute infection, displaying 1.8 and 1 log CFU reduction with respect to the untreated and early controls, respectively. It also made an addition to the synergistic effect in combination studies with first-line TB drugs (rifampin, ethambutol, isoniazid), and enacted no inhibition of five major CYP isoforms. The synthesis of **61** employing chiral epoxide **60** [36,75,76] is shown in Scheme 6, and is representative of other compounds made within this subseries.

**Figure 15:**
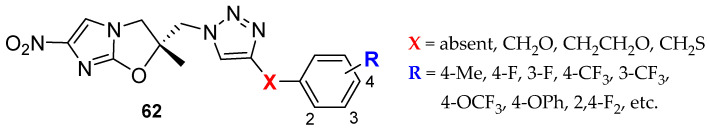
Truncated delamanid triazole analogues (**62**).

**Scheme 7:**
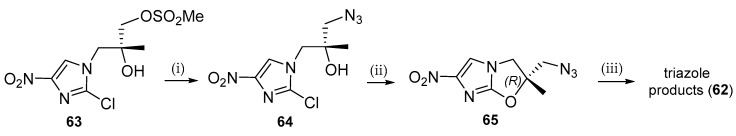
(**i**) NaN_3_, DMF, TBAB, 80 °C; (**ii**) Cs_2_CO_3_, DMF, < 15 °C–rt; (**iii**) aryl alkyne, CuSO_4_, Na ascorbate, 50% aq. *t*-BuOH, rt.

A follow up patent disclosure by the same group gives the synthesis of a small series of triazoles (**62**), in which the proximal phenyl group of **56** above has been excised in an apparent attempt to improve the physicochemical properties (lower clogP, increase aqueous solubility) (Figure 15) [83]. Target compounds were prepared as shown in Scheme 7, utilizing known chiral mesylate **63** [76] to provide novel bicyclic azide **64** for use in click reactions. The MIC values against the same strains of *M.tb* showed inferior potency for almost all analogues, underscoring the necessity of the proximal phenyl ring of **56** for good activity. No solubility data were given.

**Figure 16:**
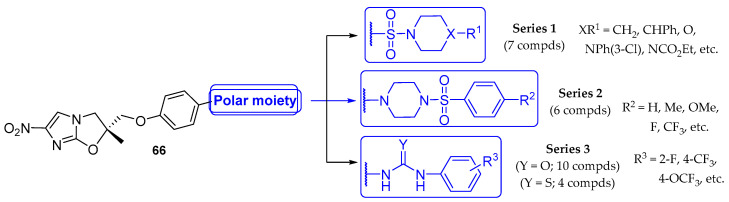
6-Nitroimidazooxazoles (**66**) with solubilizing polar moieties.

In spite of its demonstrated good in vitro and in vivo profile, compound **61** above still possesses poor physicochemical properties with high lipophilicity and measured poor aqueous solubility. This led the Indian group to design a follow-up series to address these issues [31]. Thus, compounds **66** (Figure 16) with polar functionalities appended to the 6-nitroimidazooxazole methylenephenoxy core, incorporating sulfonamide, urea and thiourea linkers, were synthesized. These were distributed across three subseries (Series 1–3). For compounds with MIC values ≤ 0.5 µg/mL vs. *M.tb*, six showed enhanced aqueous solubility. These were further tested against resistant (rifampin-resistant and MDR) and dormant strains of *M.tb*. Based on its MIC (0.37 µg/mL vs. *M.tb*), the results from secondary in vitro assays and its excellent aqueous solubility (20 µg/mL at pH 7), compound **66** (Series 1, XR^1^ = O) was chosen for further evaluation. It displayed high microsomal stability, favorable oral pharmacokinetics and showed significant log CFU reductions in an intranasal mouse model of acute infection (1.3 relative to early control over seven days, and 1.6 relative to untreated control).

**Figure 17:**
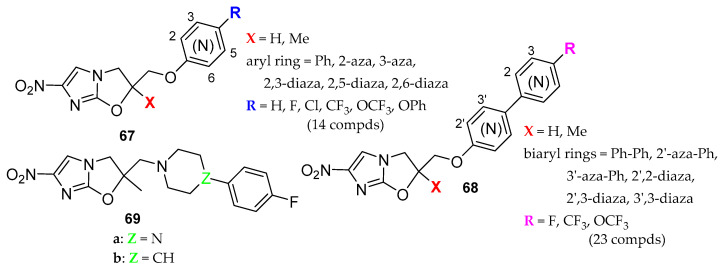
6-Nitroimidazooxazoles (**67**–**69**) for visceral leishmaniasis.

As part of an earlier program to identify a backup for the clinical trial agent pretomanid, phenotypic screening of a subset of 6-nitroimidazooxazole derivatives against kinetoplastid diseases unexpectedly led to the identification of DDNI-VL-2098 (**40**; Figure 11) as a potential first-in-class drug candidate for visceral leishmaniasis [84]. It entered into unsuccessful early stage clinical trials, with studies terminated in early 2015. Additional work was then conducted to delineate its essential structural features, with a primary goal of having an improved physicochemical/pharmacological/safety profile without compromising activity against VL [85]. The principal approach was through enhancing aqueous solubility by reducing lipophilicity (using cLopP predictions) and incorporating arylated cyclic amines (piperidine, piperazine), known bioisosteres for biaryl moieties [57], which provide for improved solubility via salt formation as well as the disruption of molecular planarity and symmetry [86]. Toward that end, 61 new analogues, most racemic, representing 13 subclasses were synthesized, incorporating modifications of the C-2 aryloxy side chain of **40**, or variations of the 6-nitroimidazole portion of the bicyclic core. The major subclasses are shown in Figure 17 with the replacement of phenyl rings by pyridine (**67**, **68** subclasses) or bioisosteric replacement (**69**). The best lead from the new library was the (*S*)-enantiomer of phenylpyridine **68** (X = H, 3′-aza, R = F), displaying high aqueous solubility at low pH, acceptable metabolic stability toward human liver microsomes, and excellent in vivo efficacy in a mouse model of acute *Leishmania donovani* infection, surpassing that of the original preclinical lead **40**. The library was also examined against TB using the in vitro assays described above. A range of MICs (0.018 to > 128 µM vs. MABA; 2.3 to > 128 µM vs. LORA) was observed. Oral dosing of **40** (MIC 0.046 µM vs. MABA; 5.9 µM vs. LORA) in a mouse model of acute TB infection showed efficacy similar to that of rifampin and superior to pretomanid, but much less active than delamanid in a comparable *M.tb* infection model, as previously reported [75].

**Figure 18:**
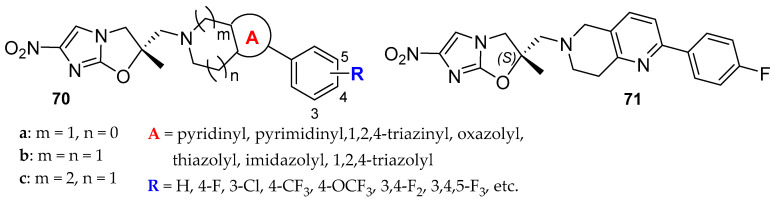
Delamanid analogues **70a**–**c** and **71** with fused bi-heterocyclic linkers.

Chinese investigators reported in a patent [87] and journal article [30] the synthesis and evaluation of 40 new analogues of delamanid, with the goal of uncovering candidates with enhanced aqueous solubility and equivalent or better safety and antimycobacterial activity. Their strategy involved the replacement of the phenoxy linker off the 6-nitroimidazooxazole core of delamanid with a 5- to 7-membered saturated aza ring (pyrrolidine, piperidine, azepane) fused to a 5- or 6-membered heteroaryl ring (A) while retaining the terminal phenyl moiety (**70a**–**c**; Figure 18). The journal article focuses on the piperidine (**70b**) series with 40 analogues, in which ring A incorporates 10 different heterocycles. All the new compounds were more hydrophilic than delamanid, and several heterocyclic series showed excellent MICs against the sensitive *M. bovis* pathogen. The most potent compounds were from the tetrahydronaphthyridine-linked nitroimidazole series. The further testing of these revealed excellent antimycobacterial activity against both replicating (MABA) and non-replicating (LORA) *M.tb*, and low cytotoxicity. The best compound (**71**) demonstrated nearly equivalent activity to delamanid in these assays, with MABA <0.03 µg/mL and LORA 4.83 µg/mL, dramatically improved aqueous solubility at pH 2 and 6.5, and better permeability, with a more favorable MDCK–MDR1 efflux ratio.

**Figure 19:**
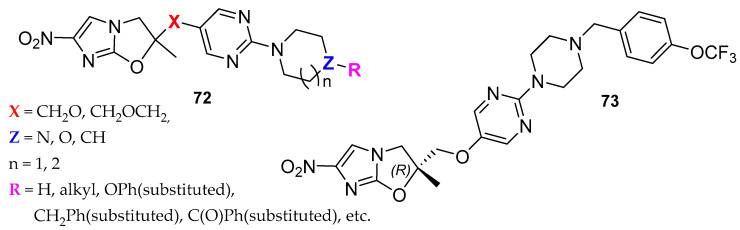
Delamanid analogues **72** and **73** with pyrimidine replacement for linking phenyl ring.

A patent application by Medshine discloses the synthesis of a class of compounds (**72**; Figure 19) related to delamanid, in which the proximal phenyl ring has been replaced by pyrimidine [88]. Synthetic details are given for the preparation of 56 analogues, either from racemic or chiral building blocks. For racemic mixtures, preparative chiral HPLC was used to separate enantiomers. From this library, MIC and cytotoxicity data were generated for 35 compounds. One compound (**73**) gave MIC values of < 0.03 µg/mL and > 32 µg/mL, respectively, vs. MABA and LORA, and IC_50_ > 32 µg/mL in Vero cells. Further evaluation showed better kinetic solubility, permeability (MDCK–MDR1 efflux ratio of 1.27) and mouse pharmacokinetics relative to delamanid.

**Scheme 8:**
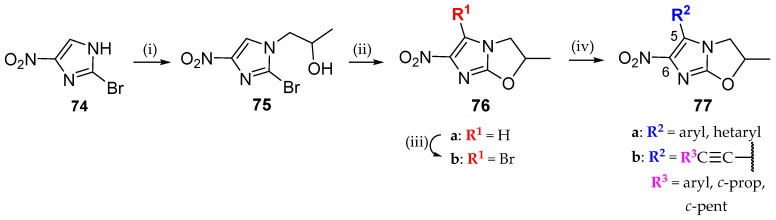
(**i**) propylene oxide, DIPEA, MW, 100 °C; (**ii**) NaH, DMF, 0 °C; (**iii**) NBS, CH_3_CN, rt; (**iv**). for **77a**: aryl boronic acid, Na_2_CO_3_, Pd(PPh_3_)_4_, DME/H_2_O (3:1), MW, 100 °C; for **77b**: alkynes, CuI, Pd(PPh_3_)_2_Cl_2_, Et_3_N, DMF, rt.

French investigators synthesized 21 simplified racemic 5-substituted 6-nitroimidazooxazole derivatives and evaluated them for their in vitro antileishmanial (*L. donovani*) and antitrypanosomal (*T. cruzi*) properties (Scheme 8) [89]. Palladium-catalyzed cross-coupling reactions were used to introduce aryl/heteroaryl and ethynyl aryl substituents (**77a** and **77b**, respectively) into the 5-position via an improved process to make 5-bromo precursor **75**. Compounds **77a** were inactive (IC_50_ > 10 µM), whereas the ethynyl aryl series **77b** showed better activity against both parasites. Against *T*. *cruzi*, **77b** (R^3^ = 3-MeO-C_6_H_4_) had IC_50_ = 0.92 µM vs. 2.31 µM for the reference drug benznidazole.

## 4. Scaffold Modifications to Bicylic Nitroimidazoles

**Scheme 9:**
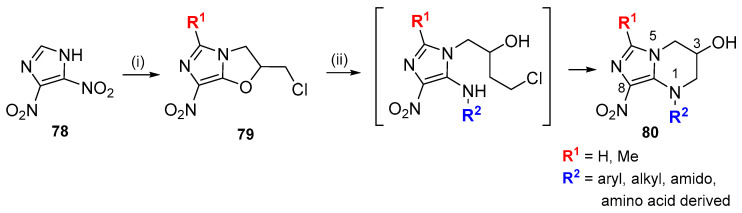
(**i**) (±)-epichlorohydrin; (**ii**) primary amine, K_2_CO_3_, EtOH, heat.

Polish workers synthesized a series of compounds (**80**), incorporating substituents off an 8-nitro-1,2,3,4-tetrahydroimidazo[1,5-*a*]pyrimidine core, in order to explore an alternative pharmacophore of pretomanid (Scheme 9) [90]. Starting from intermediate **79**, derived from condensation of **78** with racemic epichlorohydrin [91], 28 analogues were made with R^1^ = H or Me and R^2^ with side chains representing a range of lipophilicities. The latter were drawn from aromatic and aliphatic amines, and amino acids. A subset was tested against the sensitive *M.tb* and four drug-resistant strains, with all showing MIC values > 25 µg/mL.

**Scheme 10:**
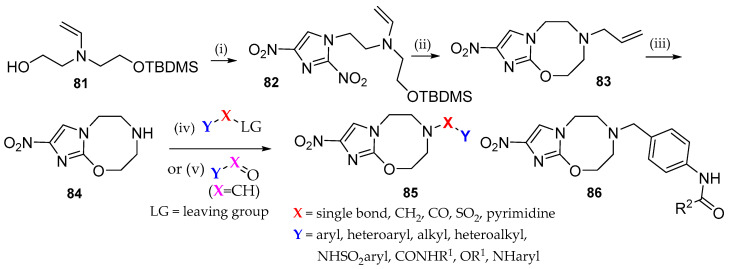
(**i**) 2,4-dinitro-1*H*-imidazole, PPh_3_, DIAD, THF, rt; (**ii**) TBAF, rt; (**iii**) 1-chloromethyl chloroformate, DCE, reflux, then MeOH, reflux; (**iv**) K_2_CO_3_, CH_3_CN:H_2_O (1:3), rt–60 °C; (**v**) NaBH(OAc)_3_ or NaBH_4_, rt.

South African researchers synthesized a large series of 9-nitro-3,4,5,6-tetrahydro-2*H*-imidazo[2,1-*b*][1,3,6]oxadiazocines (**85**; Scheme 10) with a wide range of substituents off the 8-membered ring nitrogen [92]. Two closely related synthetic pathways were used, with the dominant one shown in Scheme 10. Most analogues had X = CH_2_ and Y = aryl, heteroaryl and biaryl variants thereof. Using the MABA assay, the most potent compounds (MIC = 0.16–0.64 uM) were a subset of amides **86**.

**Figure 20:**
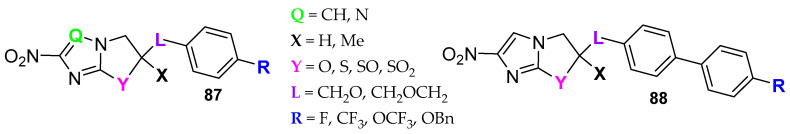
New leads (**87**, **88**) against TB and neglected tropical diseases.

The New Zealand group investigated the unexplored 6-nitro-2,3-dihydroimidazo[2,1-*b*][1,3]-thiazoles, and tested them against oxazole congeners for new leads against TB and neglected tropical diseases [93]. In total, 41 new and 11 known target compounds, most as racemates, were synthesized across several structural variations, with the major classes (**87**, **88**) shown in Figure 20. Equivalent examples with Y = O or S provided broadly comparable MICs, with 2-methyl substitution and extended aryloxymethyl side chains (**88**; L = CH_2_O) preferred for tuberculosis. However, S-oxidized thiazoles were ineffective. Favorable microsomal stability data for a biaryl thiazole (**88**: X = Me, Y = S, L = CH_2_O, R = OCF_3_) led to its assessment in an acute *M.tb* mouse model, alongside the corresponding oxazole (Y = O), but the latter proved to be more efficacious. In vitro screening against kinetoplastid diseases revealed that 6-nitroimidazothiazoles were inactive versus leishmaniasis, but showed interesting activity, superior to that of the 6-nitroimidazooxazoles, against *T. cruzi*, the protozoan parasite responsible for Chagas disease. Overall, racemic ‘‘thio-delamanid” (ring oxygen of **3** replaced by sulfur) was regarded as the best lead for use against this disease.

**Figure 21:**
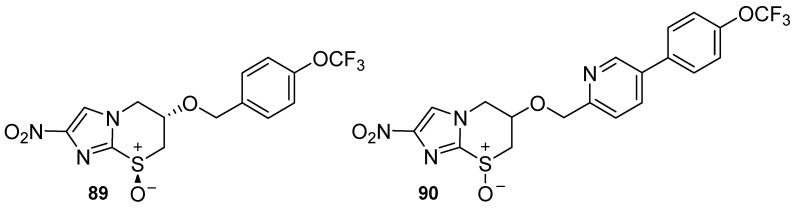
New leads to treat African trypanosomiasis.

Another campaign by the New Zealand group screened a 900-compound library for new molecular leads against human African trypanosomiasis (HAT; “African sleeping sickness”) [94]. Potent hits included 2-nitro-6,7-dihydro-5*H*-imidazo[2,1-b][1,3]thiazine 8-oxides, which displayed good metabolic stability and excellent cell permeability. Following comprehensive mouse pharmacokinetic assessments on four hits, and determination of the most active chiral form, a thiazine oxide counterpart of pretomanid (**89**; Figure 21) was identified as the best lead. The C-6 configuration of **89** was later firmly established via a known chiral synthesis [41]. With once daily oral dosing, this compound delivered complete cures in an acute infection mouse model of HAT, and increased survival times in a stage 2 model (although cure rates were inadequate), implying the need for more prolonged CNS exposure. In additional SAR studies, antitrypanosomal activity was reduced by the removal of the benzylic methylene, but enhanced through a phenylpyridine-based side chain. The best compound, **90**, a mixture of two racemic diastereomers, enhanced potency for growth inhibition against four tested trypanosomiasis subspecies, while broadly retaining other essential properties, providing important direction for future work. No test data were reported against *M.tb* for any new compounds of this study.

## 5. Computed Properties

A balance between lipophilicity and aqueous solubility is a key parameter that influences success in a standard oral, low-dose drug discovery program, and provides valuable information regarding a compound’s potential to be a drug. As mentioned in the Introduction, both pretomanid (**2**) and delamanid (**3**) are lipophilic in nature, and poorly water soluble. Toward assessing drug-like properties related to oral absorption for these agents, as well as clinically investigated ones (**4**, **40**, **50**) and selected compounds considered for advanced studies (**10**, **12**, **53**), computed properties that address these two areas are shown in Table 1. MICs vs. *M.tb* under aerobic conditions are also given for comparative purposes. A measure of lipophilicity is provided by cLogP and aqueous solubility by a solubility forecast index (SFI) calculator derived from cLogD_7.4_ information [95]. Derived cLogP values span 3.78–6.46, with DNDI-0690 (**50**) and delamanid bookmarking the range, respectively, and all except for **50** approaching or exceeding the “rule of 5” for oral agents [96]. With regard to aqueous solubility, clogD_7.4_ values, together with the number of aromatic rings in a given molecule, provide a simple, useful guide for the potential solubility of particular molecules with values ≤ 6 predicting for good aqueous solubility. Utilizing the SFI calculator, all compounds in Table 1 (SFI of 6.05–9.14) exceed the cutoff, with only compound **10** slightly above the threshold. The SFI calculations are reasonably predictive of the measured kinetic aqueous solubility for each compound. Calculations for rifampin and bedaquiline, representative of two other mechanistic classes of TB drugs, are provided for comparison.

## 6. Syntheses of Clinical Agents

### 6.1. Pretomanid (PA-824; **2**)

**Scheme 11:**
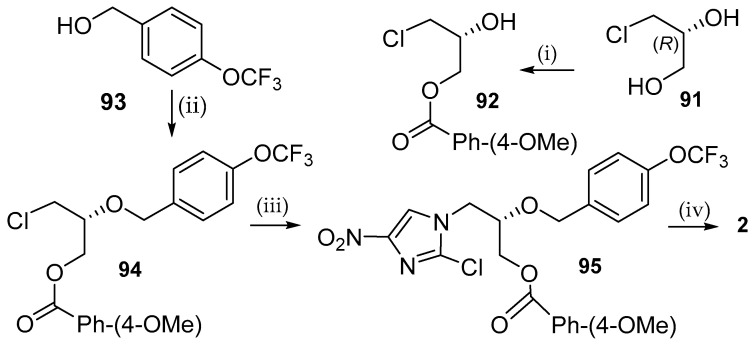
(**i**) imidazole, 4-methoxybenzoyl chloride, DCM, 0 °C–rt; (**ii**) **93**, NaH, Cl_3_CCN, TBME, 0 °C–rt; then **92**, Triflic acid, DCM, 0 °C–rt; (**iii**) 2-chloro-4-nitro-1*H*-imidazole, K_2_CO_3_/NaI (1: 1), DMF, 120 °C; (**iv**) KOH, MeOH, 0 °C–rt.

The initial synthesis of pretomanid was conducted on a laboratory scale (0.015 mol), employing the potentially explosive monomer 2,4-dinitro-1*H*-imidazole and requiring several chromatographic purifications [38]. Some of the steps in this synthesis were adapted as solventless reactions on a small scale, leading to an almost tripling of the yield [98]. A synthesis by Princeton University chemists (Scheme 11) provided significant improvements over the original route [99]. It hinged on the direct, convergent coupling of 2-chloro-4-nitro-1*H*-imidazole, a suitably safe alternative to 2,4-dinitro-1*H*-imidazole, and an appropriately functionalized glycidol (**94**) derived from the cheap building block (*R*)-3-chloro-1,2-propanediol (**91**). The overall route was executed on a 0.01 mole scale, with two chromatographic purifications in 16% overall yield and 100% enantiopurity.

**Scheme 12:**
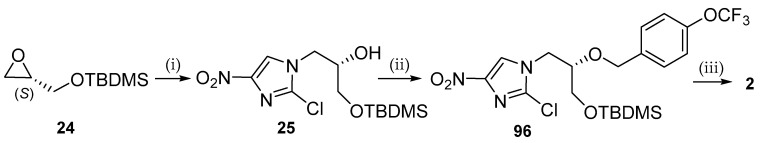
(**i**) 2-chloro-4-nitro-1*H*-imidazole, EtOH, K_2_CO_3_, 70 °C; (**ii**) 4-(trifluoromethoxy)benzyl bromide, NaH, *n*-Bu_4_NI, DMF, 0 °C; (**iii**) (a) TBAF, THF, rt; (b) KOH, MeOH, 0 °C–rt. (c) recrystallization from IPA/cyclohexane (1:2).

A patent application from Hetero Research Foundation in India outlines a similar strategy, but on a process scale (Scheme 12) [100]. Starting with the (*S*)-glycidol TBDMS ether (**24**), the 6- (trifluoromethoxy)benzyl moiety was incorporated early to provide intermediate **96**, which was then deprotected and cyclized to give protomanid (**2**). This short synthesis was executed on a 0.68 mole scale and provided **2** in 19% overall yield and high chemical purity (99.9%), following a final recrystallization. The synthesis has the attraction of brevity, but suffers from the cost of the starting chiral epoxide **24** and the instability of the TBDMS protecting group (*vide supra*, Scheme 3).

**Scheme 13:**
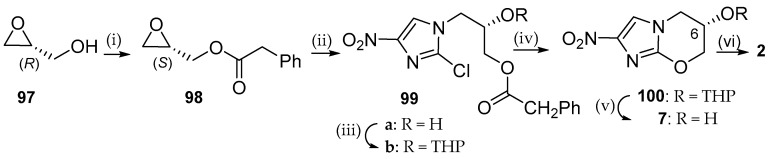
(**i**) PhCH_2_CO_2_H, EDC · HCl, DMAP, toluene, 0–5 °C; (**ii**) 2-chloro-4-nitro-1*H*-imidazole, Me_3_N, 60 °C; (**iii**) 3,4-dihydro-2*H*-pyran, PPTS, EtOAc, rt; (**iv**) KOH, MeOH, 0–5 °C; (**v**) *p*-TsOH, MeOH, rt; (**vi**) 4-(trifluoromethoxy)benzyl bromide, *t*-BuONa, DMF, 0–5 °C.

Another patent application by Indian chemists outlines a second process scale synthesis, as shown in Scheme 13 [101]. Starting with the readily available (*R*)-glycidol **97**, a series of six steps was carried out, utilizing a strategy that first incorporates the necessary framework (**99a**) of the 2- nitroimidazooxazine core. Sequential protection to **99b** followed by ester hydrolysis and ring closure gave **100**. THP ether deprotection then provided the bicyclic core (**7**), upon which the 6-(trifluoromethoxy)benzyl moiety was installed. This synthesis, while longer than that shown in Scheme 12, has the advantage of utilizing cheap reagents and provides a key intermediate **7** upon which 6-position SAR can be carried out. The scheme was carried out on a 0.55 mole scale, and provided pretomanid in 24% overall yield with high chemical (99.7%) and enantio (99.8%) purity. Both Indian routes offer simple operations in the absence of complicated purifications, making the overall processes even more cost-effective, efficient and amenable to larger scale synthesis.

**Scheme 14:**
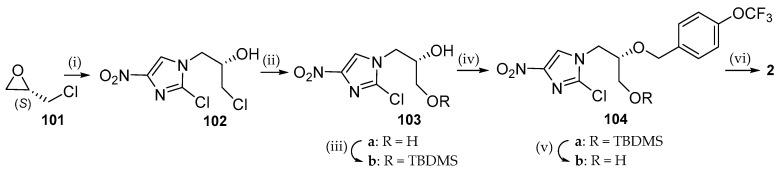
(**i**) 2-chloro-4-nitro-1*H*-imidazole, K_2_CO_3_, 70 °C; (**ii**) 2% aq. NaOH, 70 °C; (**iii**) TBDMSCl, imidazole, DMF, rt; (**iv**) 4-(trifluoromethoxy)benzyl bromide, NaH, *n*-Bu_4_NI, THF, rt; (**v**) TBAF, THF, rt; (**vi**) NaH, THF, rt; recrystallization from IPA/hexanes.

A recent paper from a Chinese group reports on a practical and efficient synthesis of pretomanid (Scheme 14) [102]. Their route, described on a 0.82 mole scale, commences with the condensation of 2-chloro-4-nitro-1*H*-imidazole with neat (*S*)-epichlorohydrin (**101**). The hydrolysis of derived chlorohydrin **102** to diol **103a** followed by TBDMS protection provides key intermediate **103b**. O-alkylation to **104a**, TBDMS ether deprotection to **104b** and intramolecular cyclization affords pure pretomanid in a 26.7% overall yield. Excellent purity (>99% by HPLC) could be achieved on a 100–200 g scale. This six-step “four-pot” route addresses the scalability issues of earlier routes, is conducted without any chromatographies, and employs cheap and readily available raw materials.

**Scheme 15:**
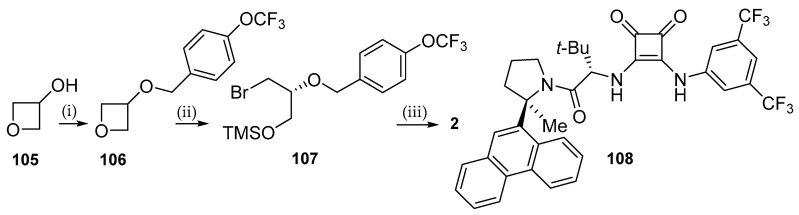
(**i**) 4-(trifluoromethoxy)benzyl bromide, NaH, 2-MeTHF, 60 °C; (**ii**) **108** (2 mol %), TMSBr, *t*-BuOMe, −80 °C; (**iii**) 2-chloro-4-nitro-1*H*-imidazole, Et_3_N, NaI, DMF, 115 °C, then NaOH, MeOH, rt.

In another recent publication, Jacobsen et al. describe the precise design of chiral squaramide catalysts, which promote highly enantioselective additions of trimethylsilyl bromide to a broad variety of 3-substituted and 3,3-disubstituted oxetanes [103]. This provides direct and general access to synthetically valuable 1,3-bromohydrin building blocks from cheap and easily-accessed achiral precursors. The enantioselective ring opening of oxetane **106**, catalyzed by squaramide catalyst **108**, was employed in a laboratory-scale synthesis of pretomanid (Scheme 15). Alkylation of 2-chloro-4-nitro-1*H*-imidazole by **107** occurred with complete regioselectivity to give an intermediate TMS ether that was deprotected in situ and cyclized to yield pretomanid (**2**). All intermediates were formed in sufficient purity to be carried forward, and only a final recrystallization was required to access analytically pure **2** in >99% enantio excess. The synthetic route avoids protecting group manipulation steps and provides access to pretomanid in just three steps.

### 6.2. Delamanid (OPC-67683; **3**)

**Scheme 16:**
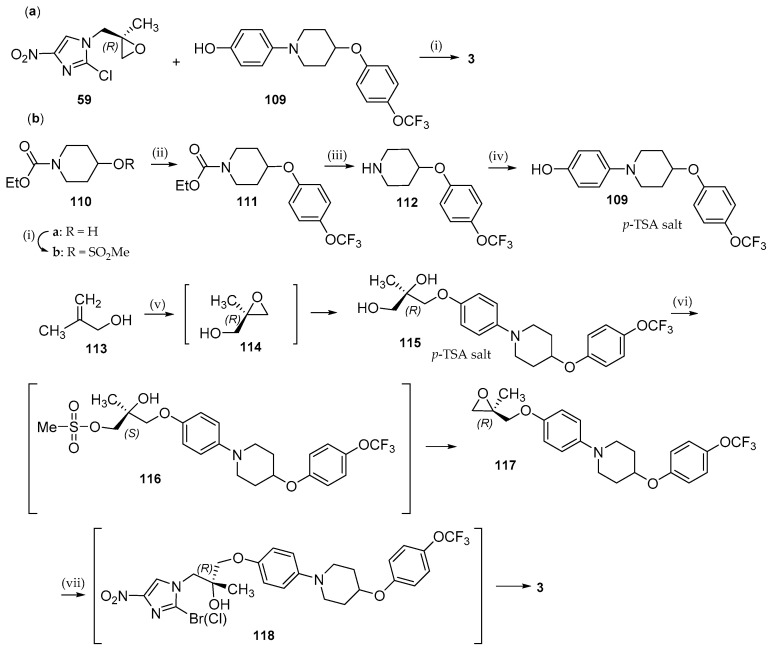
(**a**): (**i**) NaH, DMF, 50–75 °C. (**b**): (**i**) mesyl chloride, Et_3_N, toluene, rt; (**ii**) 4-(trifluoromethoxy)phenol, 25% aq KOH, 50% aq. *n*-Bu_4_NCl, toluene, 88 °C; (**iii**) KOH, toluene/EtOH (2.5:1), 98 °C; (**iv**) hydroquinone, 195–210 °C; (**v**) Ti (O-*i*-Pr)_4_, D-(-)-diisopropyl tartrate, cumene hydroperoxide, mol sieves (Zeolum A-3), toluene, −20 to −10 °C, then **109**, LiOH, DMSO, reflux, (**vi**) mesyl chloride, Et_3_N, cyclopentylmethyl ether, 0 °C, then 25% aq NaOH, water, 40–60 °C; (**vii**) 2-bromo or chloro-4-nitro-1*H*-imidazole, NaOAc, *t*-BuOAc, 100 °C, then aq NaOH, MeOH, *t*-BuOAc, 0 °C.

The original synthesis of delamanid was revealed in a 2003 patent on a mmole scale [36], followed soon thereafter on a 0.47 scale [75]. Each employed a convergent approach involving the condensation of two synthons, **59** and **109**, shown in Scheme 16a. More recent approaches utilize different retrosynthetic disconnections (Scheme 16b). The first is summarized in a review article [104], and draws from a patent in which a Buchwald reaction is carried out on suitable precursors on a 76.6 mole scale to give diol (**115**), followed by conversion to epoxide (**117**) on a 14.7 mole scale [105]. A recent patent describes the large-scale synthesis of epoxide intermediate **117,** utilizing a different approach [106]. Phenol **109** is obtained by a sequence of reactions starting from **110a*,*** with the first three reactions carried out in a single vessel on a 28 mole scale. High temperature condensation of **112** with hydroquinone on a 46 mole scale provides key phenol **109** in a four-step 79% yield. This is condensed with epoxide **114** on a 26.6 mole scale to give diol **115**, which is then mesylated (**116**) and treated with sodium hydroxide to give **117** in a three-step 80% yield. The two-step conversion of epoxide **117** to delamanid (**3**) via reaction with 2-chloro-4-nitro-1*H*-imidazole is described on a 24 mmole scale (73%) [105], and with 2-bromo-4-nitro-1*H*-imidazole on an 56 mmol scale (67.5%) [107]. A scalable route employing a sequence of reactions similar to those shown in Scheme 16b has been published by a Chinese group [108]. No kilogram scale reactions involving the bimolecular condensation of known synthons to give delamanid have been described in the literature.

### 6.3. DNDI-VL-2098 (**40**)

**Scheme 17:**
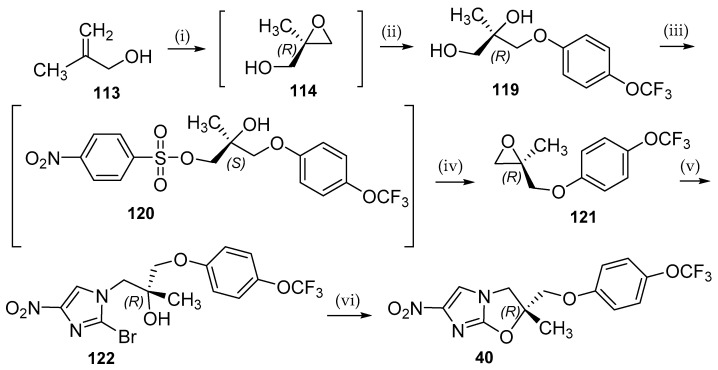
(**i**) Ti (*O*-*i*-Pr)_4_, d-(−)-diisopropyl tartrate, TBHP, mol sieves 4A, DCM, –30 to –5 °C; (**ii**) 4-(trifluoromethoxy)phenol, K_2_CO_3_, MeOH, 60 °C; (**iii**) 4-nitrobenzenesulfonyl chloride, Et_3_N, DCM, rt; (**iv**) 12.5 M aq NaOH/DCM, 15 °C; (**v**) 2-bromo-4-nitro-1*H*-imidazole, *i*-Pr_2_NEt, 110 °C; (**vi**) K_2_CO_3_, DMF, 90 °C.

Satam et al. developed a process suitable for a kilogram-scale synthesis of DNDI-VL-2098 (**40**) [109], which was in clinical trials until early 2015 for the treatment of visceral leishmaniasis. The six-step synthesis is shown in Scheme 17, and commences with the Sharpless asymmetric epoxidation of 2-methyl-2-propen-1-ol (**113**), which required the development of a simple, safe procedure to obtain anhydrous TBHP from commercially available 70% aqueous solution. The derived water soluble epoxide **114** was not isolated, but reacted with 4-(trifluoromethoxy)phenol to give diol **119**. The selective activation of the primary alcohol with 4-nitrobenzenesulfonyl chloride provided intermediate **120**, which was cleanly converted in situ to epoxide **121** in a biphasic reaction. Condensation of this with 2-bromo-4-nitro-1*H*-imidazole [110] gave a high yield of **122**, which was then ring closed to **40**. Overall, the process was simplified by employing the in situ synthesis of some intermediates, reducing safety hazards and eliminating column chromatographic purifications. The process was conducted on a large scale to produce 5–6 kg lots of **40**, in good overall yield (19.4%) and high optical and chemical purity (99.5% each).

## 7. Conclusions and Future Perspectives

This review summarizes recent progress in the discovery and development of a new drug class of bicyclic nitroimidazoles for the treatment of tuberculosis and selected neglected tropical diseases. Most of the reported work highlights SAR campaigns to uncover second-generation drugs over the last decade within the 2-nitroimidazooxazine and 6-nitroimidazooxazole subclasses, represented by the recently approved drugs pretomanid (**2**) and delamanid (**3**), respectively. In addition, the synthesis of scaffold variations within these subclasses has provided new chemical matter for additional directions of research, especially with respect to new directions for neglected tropical diseases. Several agents with excellent in vitro and in vivo profiles have emerged, including TBA-354 (**4**), which was enrolled in an unsuccessful Phase 1 clinical trial for the treatment of tuberculosis, and DNDI-0690 (**50**), currently in Phase I trials for visceral leishmaniasis.

A Scifinder^®^ search revealed that there are ~2400 unique 2-nitroimidazooxacine structures disclosed in manuscripts and patents dating back to 1997, with ~1800 of these from 2011 to mid-2020. A high proportion of these are target compounds made for biological evaluation. For 6-nitroimidazooxazoles, the corresponding numbers dating back to 1989 are ~3500 and ~500, respectively. While extensive SAR campaigns carried out by various laboratories world-wide uncovered a number of lead compounds with good preclinical attributes, such as those shown in Table 1, none show data that are compelling enough to supplant pretomanid (**2**) or delamanid (**3**), leading to the conclusion that further alternative SAR directions within either subclass of bicyclic nitroimidazoles will unlikely yield comparable or superior drugs for the treatment of TB. However, with a high medical need for better drugs to treat selected neglected diseases, further SAR efforts with bicyclic nitroimidazoles and congeners seem justified.

Potential future campaigns for a second generation of TB drug(s) within the bicyclic nitroimidazole class will be driven by any emergence of resistance to pretomanid and delamanid. A structure-based design approach, based on a co-crystal structure of either pretomanid or delamanid, or an analogue, with deazaflavin-dependent nitro-reductase (Ddn), would offer an attractive alternative to current “phenotypic” SAR approaches. Previous attempts at this have been unsuccessful [111]. However, new technologies for protein production, x-ray crystallography with very small crystals and cryo electron microscopy are poised to overcome previous hurdles, and expand the impact of structural biology on drug discovery over the coming decade [112].

## Figures and Tables

**Table 1:** Computed properties of selected bicyclic nitroimidazoles.

#	Structure	MIC *M.tb. ^a^*	MW	TPSA (Å) *^b^*	cLogP *^b^*	cLogD_7.4_ *^b^*	SFI *^c^*	Aq. Sol.
**2**	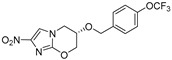	0.05	359.26	88.65	4.50	4.14	6.14	19 [47] *^d^* 53 µM
**3**	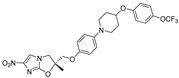	0.01	534.49	101.12	6.46	6.14	9.14	0.31 [85] *^d^* 0.6 µM
**4**	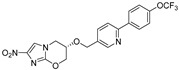	0.05	436.35	101.54	5.27	4.95	7.95	2.3 [47] *^d^* 5.3 µM
**10**	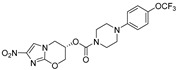	0.03	457.37	112.20	4.75	4.05	6.05	17 [55] *^d^* 37 µM
**12**	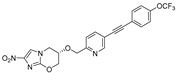	0.02	460.37	101.54	5.32	5.13	8.13	0.36 [57] *^d^* 0.8 µM
**40**	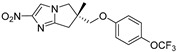	0.05	357.29	79.42	4.72	4.19	6.19	2.4 [85] *^d^* 6.7 µM
**50**	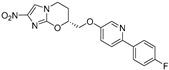	0.11	370.34	92.31	3.78	3.69	6.69	0.45 [68] *^d^* 1.2 µM
**53**	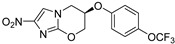	31	345.23	88.65	4.50	4.10	6.10	12 [72] *^d^* 35 µM
**rifampin**		0.03	822.95	220.15	3.76	2.75	2.75	2.5 mg/mL *^e^* 3.0 mM
**bedaquiline**		0.04	554.53	32.70	7.24	5.82	10.82	<0.06 µg/mL [97] *^f^* <0.1 µM

*^a^* Minimum inhibitory concentration (µM) determined under aerobic (MABA) conditions. *^b^* ChemAxon calculator, MarvinSketch 19.18.0, 2019. *^c^* Solubility Forecast Index (SFI) = cLogD_7.4_ + #aromatic rings. Values ≤ 6 predict for good aqueous solubility [95]. *^d^* Kinetic solubility (µg/mL) at pH 7.0 and 20 °C [47]. *^e^* pH 7.3. *^f^* pH 7.4.
